# Ten year anniversary of AMP AD: Enabling a Precision Medicine Approach to Target and Biomarker Discovery for Alzheimer’s Disease

**DOI:** 10.1002/alz.086464

**Published:** 2025-01-03

**Authors:** Nilüfer Ertekin‐Taner, Suzana S Petanceska

**Affiliations:** ^1^ Mayo Clinic, Jacksonville, FL USA; ^2^ National Institute on Aging, Bethesda, MD USA

## Abstract

**Background:**

Alzheimer’s disease (AD) is complex and multifactorial. Precision medicine approaches are needed to capture the basis of heterogeneity in AD pathogenesis, clinical presentation and neuropathology. Large‐scale molecular, deep phenotypic and exposomal data necessary to enable precision medicine research requires team‐based, interdisciplinary programs. Accelerating Medicines Partnership in Alzheimer’s Disease (AMP‐AD) was launched in 2014 with the goals to characterize disease heterogeneity and discover precision‐medicine therapeutic targets and biomarkers for AD and related disorders (ADRD).

**Method:**

AMP‐AD consortium, comprising eight teams addresses the barriers that historically limited therapeutic target and biomarker discovery in ADRD by characterizing the molecular disease complexity in deeply phenotyped human biospecimens and experimental disease models. Over the past decade, AMP‐AD teams generated multi‐omics data in ∼2,000 human brain samples from largely non‐Hispanic white donors and from >600 African American and Latin American donors. AMP‐AD datasets, including bulk and single cell molecular profiles from brain and peripheral tissues are analyzed using innovative analytic approaches generated and applied by these teams. Findings are validated with a broad array of cross‐species experimental platforms including human iPSC, fly and rodent models. These outcomes are integrated with deep phenotypic and exposomal measures for precision medicine discoveries and broadly shared.

**Result:**

AMP‐AD generates high‐dimensional molecular data and applies novel systems biology approaches bringing new insights into ADRD. AMP‐AD teams discovered molecular subtypes of AD and characterized the cell‐type specificity of these molecular changes. Discovery of disease signatures in brain and peripheral tissues enabled nominations of precision medicine therapeutic targets and biomarkers. Over 600 targets, annotated with human and cross‐species data, are shared with the research community on the Agora Platform of AD Knowledge portal that also houses all research data and outputs for rapid and responsible dissemination in a centralized manner.

**Conclusion:**

First decade of AMP‐AD led to unprecedented data and knowledge for rigorous and reproducible ADRD research, accelerated the delivery of many new disease insights, and expanded the target and biomarker landscape. Future directions include discovery of subtypes for disease specificity and progression, contextualized by multi‐ethnic, molecular, exposomal data and validation platforms well‐integrated into therapeutic development pipelines to deliver new cures to ADRD.

